# Knowledge and Use of Herbal Medicine Among Urban Adolescents and Young Adults in Western Mexico: Family Transmission, Social Media Exposure, and Associated Factors

**DOI:** 10.3390/healthcare14142161

**Published:** 2026-07-17

**Authors:** Gustavo A. Hernández-Fuentes, Emmanuel Vallejo-Tapia, Osiris G. Delgado-Enciso, Mario A. Alcalá-Pérez, Uriel Díaz-Llerenas, Mercedes Fuentes-Murguia, Nibardo Cobian-Gutierrez, Juan M. Sánchez-Galindo, Carmen A. Sanchez-Ramirez, José Guzmán-Esquivel, Fabian Rojas-Larios, Ángel A. Ramos-Organillo, Ariana Cabrera-Licona, Iván Delgado-Enciso

**Affiliations:** 1 Department of Molecular Medicine, School of Medicine, University of Colima, Colima 28040, Mexico; odelgado3@ucol.mx (O.G.D.-E.); fuentes_murguia@ucol.mx (M.F.-M.); juanmanuelsg02@gmail.com (J.M.S.-G.); carmen_sanchez@ucol.mx (C.A.S.-R.); frojas@ucol.mx (F.R.-L.); arianacabrera267@gmail.com (A.C.-L.); 2 State Cancerology Institute of Colima, Health Services of the Mexican Social Security Institute for Welfare (IMSS-BIENESTAR), Colima 28085, Mexico; 3 Faculty of Chemical Sciences, University of Colima, Coquimatlan 28400, Mexico; evallejo0@ucol.mx (E.V.-T.); aaramos@ucol.mx (Á.A.R.-O.); 4 Molecular Medicine Laboratory, Academic Unit of Human Medicine and Health Sciences, Autonomous University of Zacatecas, Zacatecas 98160, Mexico; marioalcalaperez@uaz.edu.mx (M.A.A.-P.); urieldiazllerenas@gmail.com (U.D.-L.); 5 Tecnológico Nacional de México, Campus Colima, Villa de Alvarez 28976, Mexico; ncobian01@hotmail.com; 6 Clinical Epidemiology Research Unit, Mexican Institute of Social Security, Villa de Alvarez 28984, Mexico; jose.esquivel@imss.gob.mx; 7 Robert Stempel College of Public Health and Social Work, Florida International University, Miami, FL 33199, USA

**Keywords:** complementary medicine, herbal medicine, adolescent health, traditional medicine, medical pluralism, health communication, digital media

## Abstract

**Highlights:**

**What are the main findings?**

- Herbal medicine was used by 36.8% of urban adolescents and young adults, and all users reported previous experience using herbal medicine together with conventional medical treatment, suggesting coexistence of both therapeutic approaches.
- Family-based recommendation was associated with herbal medicine use (OR = 8.04) and recommendation behavior (OR = 3.70), whereas social media exposure was associated only with self-reported herbal medicine knowledge (OR = 2.97).

**What are the implications of the main findings?**

- Herbal medicine use should be considered during routine healthcare encounters with adolescents and young adults because many individuals report experience using herbal medicine alongside conventional medical treatment.
- Family-based recommendation appears to play a central role in herbal medicine-related behaviors, whereas social media may primarily contribute to knowledge acquisition, supporting the development of culturally sensitive health communication strategies.

**Abstract:**

**Background/Objectives**: Herbal medicine is one of the most widely used forms of complementary medicine worldwide. Although traditionally associated with rural populations and older generations, its use among urban adolescents and young adults and the factors associated with its use remain insufficiently understood. This study aimed to evaluate herbal medicine knowledge, use, recommendation practices, and their associations with family-based recommendation, social media exposure, and previous experience using herbal medicine together with conventional medical treatment among urban adolescents and young adults in western Mexico. **Methods**: A cross-sectional study was conducted among 144 urban high school students in western Mexico. A structured questionnaire was used to assess sociodemographic characteristics, herbal medicine knowledge, use, recommendation practices, family-based recommendation, social media exposure, and self-reported concurrent use with conventional medical treatment. Bivariate and multivariable logistic regression analyses were performed to identify factors independently associated with herbal medicine use, recommendation, and self-reported knowledge. **Results**: Herbal medicine use was reported by 36.8% of participants, whereas 31.9% reported knowing what herbal medicine is. Family-based recommendation was independently associated with herbal medicine use (OR = 8.04; 95% CI: 2.99–21.61; *p* < 0.001), followed by self-reported herbal medicine knowledge (OR = 4.56; 95% CI: 1.97–10.58; *p* < 0.001). Recommendation behavior was independently associated with family-based recommendation (OR = 3.70; 95% CI: 1.45–9.44; *p* = 0.006) and previous herbal medicine use (OR = 4.16; 95% CI: 1.75–9.93; *p* = 0.001). Among herbal medicine users, all participants reported previous experience using herbal medicine together with conventional medical treatment, suggesting the coexistence of both therapeutic approaches within this study population. Social media exposure was associated with self-reported herbal medicine knowledge (OR = 2.97; 95% CI: 1.06–8.27; *p* = 0.038) but was not associated with herbal medicine use or recommendation. **Conclusions**: Among this study population, herbal medicine was commonly reported and appeared to be part of complementary healthcare practices among urban adolescents and young adults. Family-based recommendation was independently associated with herbal medicine use and recommendation, whereas social media exposure was associated primarily with self-reported knowledge rather than behavioral outcomes. These findings highlight the importance of considering herbal medicine use during clinical communication and adolescent health education while recognizing the coexistence of herbal and conventional healthcare practices.

## 1. Introduction

Herbal medicine (HM) is one of the most widely used forms of traditional medicine and is frequently incorporated into complementary medicine worldwide [1,2,3,4]. Increasingly, herbal medicine is recognized as a component of complementary medicine, particularly when used alongside conventional medical treatments to address common health concerns [5] and improve overall well-being [6,7,8]. Although herbal medicine has traditionally been associated with rural populations and older generations, increasing evidence indicates that its use also persists among urban adolescents and young adults [5,6,7,8]. Rather than being displaced by modernization, urbanization, or formal education, herbal medicine often coexists with biomedical care [9,10,11], reflecting contemporary patterns of complementary medicine use and health-related decision-making [2,12,13].

Among adolescents and young adults, herbal medicine is commonly used to manage everyday health complaints [14], including digestive disorders, respiratory symptoms, musculoskeletal discomfort, anxiety, and sleep disturbances [14,15]. In many settings, these practices are not used as alternatives to conventional medicine but rather as complementary approaches integrated into broader healthcare practices. Understanding the factors associated with herbal medicine knowledge, use, and recommendation among younger populations is therefore relevant for public health and complementary medicine research [16,17].

Notably, most empirical evidence on HM use and knowledge transmission has been generated in adult populations, patients, or healthcare professionals [12,16,17]. Studies conducted in hospital settings in western Mexico have shown that HM knowledge circulates not only among patients but also through healthcare personnel, contributing to its visibility within clinical contexts [12]. However, these findings largely reflect adult-centered or institutional environments and do not fully capture the dynamics operating in younger, non-clinical populations [18,19].

In parallel, digital social networks and contemporary communication platforms have emerged as relevant spaces for the circulation and reconfiguration of traditional herbal knowledge [20]. Rather than replacing familial transmission, these platforms may modify processes of knowledge acquisition and validation, particularly in relation to traditionally derived products presented in modernized or commercialized formats [21]. This convergence suggests that traditional family transmission and digital communication now coexist as complementary sources of herbal medicine information [22,23,24]. Nevertheless, the relative association of these pathways remains insufficiently understood, especially in contexts where traditional medicine continues to be embedded in everyday life, such as Mexico [25,26,27].

Given the sociocultural complexity of adolescence and early adulthood, urban students represent a particularly relevant population for examining these dynamics, as they are simultaneously exposed to family traditions, formal education, biomedical discourse, and digital media [28,29]. This intersection creates a unique setting in which the maintenance and adaptation of herbal medicine within contemporary healthcare practices are likely to be particularly relevant [30].

For the purposes of this study, herbal medicine (HM) refers specifically to the use of medicinal plants for health-related purposes and is used as the primary analytical term throughout the manuscript [1,2]. Traditional medicine is used only when referring to the broader cultural and ethnomedical context in which HM is embedded. Complementary medicine refers to the use of HM alongside conventional medical care, whereas the term alternative medicine is retained only when referring to terminology from previously published instruments or cited literature [17,31,32].

Based on these considerations, the present study aimed to evaluate herbal medicine knowledge, use, recommendation, previous experience with herbal medicine together with conventional medical treatment, as well as their associations with family transmission and social media exposure among urban adolescents and young adults in western Mexico. Specifically, we examined the associations of family transmission, digital media exposure, and concurrent use with conventional medicine on herbal medicine knowledge, use, and recommendation. By distinguishing knowledge from behavioral outcomes, this study provides insight into how complementary medicine practices are preserved and integrated into the health-related decision-making of contemporary urban youth.

## 2. Materials and Methods

### 2.1. Study Design

This study employed a quantitative, observational, cross-sectional design, embedded within a broader research program focused on health behaviors and sociocultural factors among students enrolled in a semi-school-based educational model [30]. This design was selected to characterize patterns of knowledge, use, recommendation, and sociocultural transmission of herbal medicine (HM) in an urban youth population, as well as to examine associations between family influence, exposure to digital media, and the adoption and recommendation of HM practices.

The analytical framework was structured to differentiate between cognitive (knowledge) and behavioral (use and recommendation) dimensions of HM, allowing the identification of discrepancies between awareness and practice. Particular emphasis was placed on evaluating the role of interpersonal (e.g., family) and digital (e.g., social media) pathways as complementary but distinct mechanisms associated with HM-related outcomes.

Two primary outcomes were analyzed: (1) HM use and (2) recommendation behavior. Given the cross-sectional nature of the study, associations were interpreted as non-causal, acknowledging that bidirectional relationships between exposure, knowledge, and behavior are plausible.

Data were collected during the regular academic period in October 2025 at a private high school operating under a semi-school-based model with Monday-to-Friday classes, located in an urban area of Colima, Mexico.

### 2.2. Study Population

The study population consisted of students enrolled in the fourth academic term of an upper secondary semi-school-based program. All students attending regular classes during the data collection period were invited to participate. Inclusion criteria were current enrollment, presence during data collection, and completion of the informed consent procedures before participation. Participants were enrolled in an upper-secondary education program and ranged in age from 15 to 21 years. Although most respondents were adolescents, older students remained enrolled in the same educational program, reflecting the characteristics of the semi-school-based educational model.

Written informed consent was obtained from all adult participants. For participants younger than 18 years, written informed consent was obtained from their parents or legal guardians, and written assent was obtained from the participants before enrollment.

Exclusion criteria included absence during questionnaire administration, incomplete or inconsistent responses, or voluntary withdrawal [33,34]. The proportion of excluded cases was minimal (<10%) [35].

### 2.3. Data Collection and Instrument

Data were collected using a structured, self-administered questionnaire completed face-to-face under the supervision of trained research personnel. The instrument required approximately ten minutes to complete and was specifically designed to assess sociocultural mechanisms of transmission, knowledge, use, and recommendation of herbal medicine (HM) in an urban student population.

The questionnaire gathered information on sociodemographic characteristics, including age, sex, employment status, and self-reported body weight. Socioeconomic status was assessed using the 2022 AMAI classification, which categorizes households into levels ranging from A/B (high) to E (low), following nationally validated guidelines [36,37,38]. Family and cultural background were evaluated through variables such as self-identification or familial ties to indigenous communities and the highest educational level within the household, serving as proxies for intergenerational transmission of traditional knowledge.

The instrument was organized to capture four analytical domains: (1) knowledge of herbal medicine (HM), (2) lifetime HM use, (3) recommendation behavior, and (4) sources and pathways of information. Knowledge was assessed through a dichotomous question asking whether participants knew what herbal medicine was (Yes/No). Lifetime use was assessed by asking whether participants had ever used herbal medicine (Yes/No), followed by a question on frequency of use. Recommendation behavior was assessed by asking whether participants had ever recommended herbal medicine to another person (Yes/No), whereas family-based recommendation was operationalized as reporting a family member as the primary source of recommendation. Sources of information included family members, friends, neighbors, traditional healers, healthcare professionals, and other sources. Social media exposure was assessed by asking whether respondents obtained herbal medicine-related information through social media and whether they shared, liked, commented on, or otherwise interacted with such content. Among herbal medicine users, concurrent use with conventional medicine was assessed by asking whether they had ever used herbal medicine together with conventional (allopathic) treatment. The questionnaire did not distinguish whether both therapies were used simultaneously for the same health condition or at different times for different conditions.

Respondents reporting herbal medicine use were also asked about their primary reason for use and the therapeutic categories of herbal medicines used. Primary reasons for use were recorded as a single response for each participant, whereas therapeutic categories (e.g., digestive, respiratory/expectorant, relaxant/sedative, dermatological, anti-inflammatory/wound-healing, hypoglycemic/antidiabetic, immunostimulant, and emmenagogue) were assessed using a multiple-response format [39,40]. Consequently, percentages for therapeutic categories were derived from multiple-response analyses and do not sum to 100%. Unless otherwise indicated, percentages were calculated using the number of herbal medicine users as the denominator. Variables were operationalized to distinguish cognitive (knowledge) and behavioral (use and recommendation) dimensions, enabling assessment of their associations with family transmission and digital information pathways. The use of specific social media platforms (e.g., Facebook, Instagram, Snapchat, TikTok, and X) was also recorded [41].

The questionnaire was adapted from previously published surveys assessing herbal medicine knowledge, attitudes, use, sources of recommendation, and complementary medicine practices and was tailored to the objectives of the present study and the local sociocultural context [31,42]. Additional items addressing family-based transmission, concurrent use with conventional medicine, and social media exposure were incorporated based on the study objectives and current evidence on digital health communication [43,44]. Before the main study, the questionnaire was pilot-tested in 20 students to evaluate clarity, comprehensibility, cultural appropriateness, and feasibility. Minor wording modifications were introduced following participant feedback. Although the questionnaire underwent pilot testing, formal psychometric validation, including assessments of internal consistency, test–retest reliability, and construct validity, was not performed. The questionnaire items analyzed in the present study are provided in Supplementary Table S1.

### 2.4. Ethical Considerations

Participation was entirely voluntary. Written informed consent was obtained from all adult participants. For participants younger than 18 years, written informed consent was obtained from their parents or legal guardians, and written assent was obtained from the participants before enrollment. All responses were collected anonymously without any personal identifying information. The study was conducted in accordance with the Declaration of Helsinki and approved by the Research Ethics Committee of the State Cancer Institute and the University of Colima authorities (approval No. CEICANCL230317-ASEXCCC-06, 2 March 2017). The present study was performed under an Ethics Committee-approved extension of the original research protocol entitled “Relationship between Sexual Activity, Inhibited Sexual Desire, Self-Esteem, Substance Use, and Mental Health in Students”, which expanded the approved scope of data collection to include variables related to herbal medicine use among urban adolescents.

### 2.5. Statistical Analysis

Statistical analyses were performed using SPSS Statistics version 28 (IBM Corp., Armonk, NY, USA). Descriptive statistics were used to summarize all study variables. Categorical variables were expressed as frequencies and percentages, and continuous variables as means ± standard deviations. Normality was assessed using the Shapiro–Wilk test and graphical inspection.

Bivariate analyses explored crude associations between explanatory variables and two primary outcomes: (1) use of herbal medicine (HM) and (2) recommendation behavior. Associations were evaluated using Pearson’s chi-square or Fisher’s exact test, as appropriate. Crude odds ratios (OR) with 95% confidence intervals (CI) were calculated.

Given the study focus, particular attention was placed on the relationship between knowledge, use, and recommendation, as well as on the role of information sources, including family and digital media. To account for potential confounding while maintaining model stability relative to sample size, multivariable logistic regression models were constructed using a limited number of predictors selected based on statistical significance and theoretical relevance [45].

The final model for recommendation behavior included interpersonal (family and peers) and digital exposure variables. Adjusted odds ratios (aOR) with 95% CI were reported.

Goodness-of-fit was assessed using the Hosmer–Lemeshow test. Multicollinearity was evaluated by examining coefficient stability and standard errors, and no evidence of problematic multicollinearity was identified. Categories with sparse data were not combined because they represented conceptually distinct groups; instead, variables with low cell counts were interpreted cautiously and, when appropriate, described descriptively rather than emphasized in multivariable analyses. Additional exploratory models were constructed using knowledge as a dependent variable.

The sample size corresponded to the total accessible population (N = 158), yielding a final analytical sample of 144 participants (response rate: 91.1%). Although adequate for detecting moderate associations, the relatively modest sample size limited the number of predictors that could be included in the multivariable logistic regression models without increasing the risk of model overfitting and unstable parameter estimates. Therefore, variables included in the adjusted models were selected a priori based on their theoretical relevance to the study objectives and their statistical significance in the bivariate analyses, rather than adjusting for all available demographic variables. Consequently, residual confounding cannot be completely excluded, and the findings should be interpreted within this context [46,47,48,49]. To improve transparency, the observed cell frequencies underlying the multivariable logistic regression models are provided in Supplementary Table S2.

## 3. Results

### 3.1. Sociodemographic Characteristics of the Study Population

**Figure 1:**
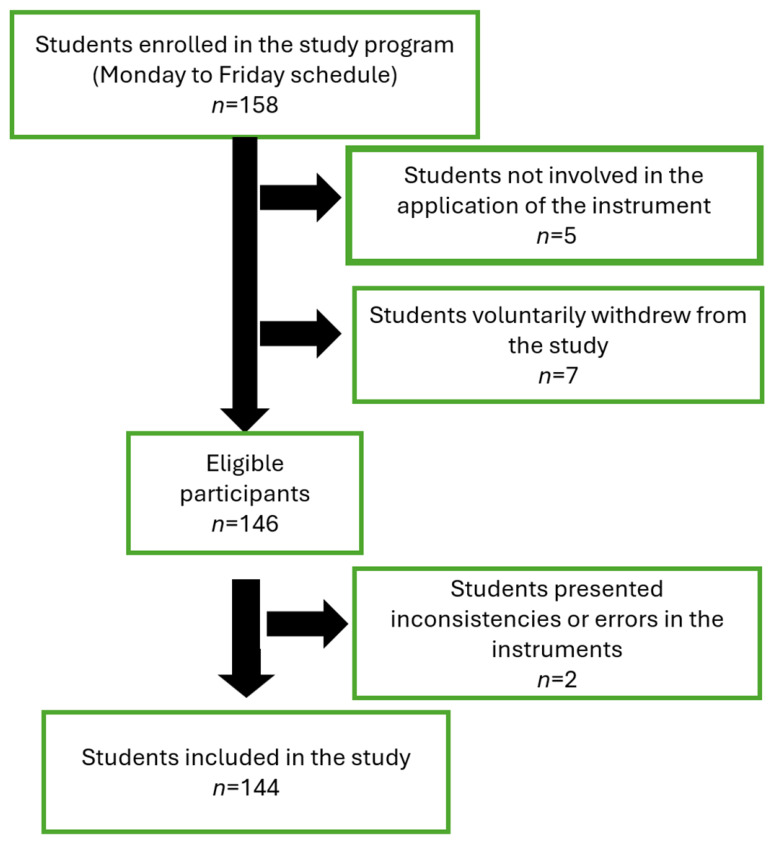
Participant Selection Flowchart: Invitation, Inclusion, and Exclusion.

From a total of 158 students enrolled in the fourth semester of the upper-secondary semi-school-based educational program (Monday-to-Friday schedule), 14 were excluded due to incomplete data according to the predefined exclusion criteria. Thus, the final analytical sample comprised 144 participants. The participant selection process is summarized in Figure 1. The sample included 54.2% females and 45.8% males. The mean age was 17.99 ± 1.31 years (median: 17; range: 15–21), with slightly higher values observed among women. The mean body mass index (BMI) of the total sample was 24.61 ± 4.97 kg/m^2^, with a median of 24.16 and a range from 13.67 to 40.40. When stratified by sex, males presented a slightly higher mean BMI (24.92 ± 4.40 kg/m^2^; median: 24.58; range: 16.98–38.06) compared with females (24.35 ± 5.43 kg/m^2^; median: 23.85; range: 13.67–40.40).

Most respondents (94.4%) reported no affiliation with an indigenous community, while 5.6% indicated some degree of affiliation or family ties. Additionally, 57.6% of students reported engaging in paid employment alongside their studies. Regarding family context, the highest educational level within households was most frequently upper secondary education (39.6%) or undergraduate studies (27.1%), although a smaller proportion reported low educational attainment (7.7% combined). Socioeconomic status showed a higher concentration in lower strata (D and E; 49.3%), with no participants classified in the highest level (A/B). Detailed data are presented in Table 1.

### 3.2. Knowledge and Use of Traditional Medicine Among Urban Students

Among the total sample, 31.9% of students reported knowing what herbal medicine is, whereas 36.8% reported having used it at least once, indicating that reported use exceeded self-reported knowledge.

Among herbal medicine users (*n* = 53), family members were the most frequently reported source of recommendation (60.4%), whereas alternative health practitioners, physicians or nurses, and herbalists accounted for smaller proportions. Social media was not reported as a direct source of recommendation (Table 2).

The main reasons for herbal medicine use were musculoskeletal pain (24.5%) and gastrointestinal discomfort (9.4%). Some participants also reported using herbal medicine in relation to chronic conditions such as cancer, hypertension, and dyslipidemia; however, these uses were described primarily as preventive or prophylactic rather than for the treatment of diagnosed conditions.

### 3.3. Coexistence of Herbal and Conventional Medicine Use

Regarding the coexistence of herbal medicine and conventional care, all students who reported using herbal medicine indicated that they combined it with allopathic treatments (100% of users), reflecting the coexistence of herbal medicine and conventional medical treatment within the context of medical pluralism in this population. In terms of frequency, 73.6% of users reported using herbal medicine at least once per week, including 15.1% who reported daily use.

### 3.4. Types and Functional Categories of Herbal Medicine Use

**Figure 2:**
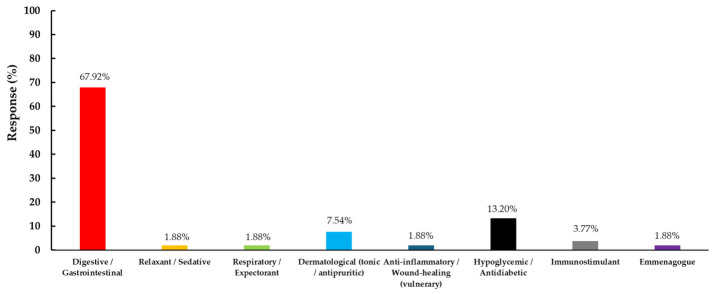
Functional categories of herbal medicine use among students. Data represents the distribution of reported uses among students who declared prior use of herbal medicine (n = 53). Categories were defined according to the primary therapeutic purpose of each reported use, including digestive/gastrointestinal, hypoglycemic/antidiabetic, dermatological (tonic/antipruritic), immunostimulant, relaxant/sedative, respiratory/expectorant, anti-inflammatory/wound-healing, and emmenagogue applications. Participants could report more than one therapeutic category; therefore, percentages were calculated based on the total number of reported uses and do not sum to 100%.

Among students who reported using herbal medicine (*n* = 53), plant-based remedies were used predominantly for digestive and gastrointestinal purposes, representing 67.9% of reported uses. Other functional categories were less frequent, including hypoglycemic or antidiabetic uses (13.2%) and dermatological applications (7.5%). Less frequently reported therapeutic categories included immunostimulant purposes (3.8%), as well as relaxant/sedative, respiratory/expectorant, anti-inflammatory or wound-healing, and emmenagogue applications, each accounting for less than 2% of responses. These results describe the distribution of therapeutic uses among herbal medicine users. To identify factors associated with herbal medicine use, subsequent analyses were performed using the entire study population (*n* = 144) (Figure 2).

### 3.5. Factors Associated with Herbal Medicine Use

Bivariate analyses were conducted to evaluate factors associated with herbal medicine use among students (Table 3). Family-based recommendations were significantly associated with herbal medicine use. Students who reported that herbal medicine was recommended by family members had significantly higher odds of use (OR = 10.71; 95% CI: 4.18–27.45; *p* < 0.001). Similarly, knowledge of herbal medicine was significantly associated with use (OR = 6.11; 95% CI: 2.84–13.15; *p* < 0.001). In contrast, obtaining health-related information from social media was not significantly associated with herbal medicine use (OR = 1.21; 95% CI: 0.52–2.83; *p* = 0.658).

The probability of herbal medicine use was evaluated using a multivariable logistic regression model including family-based recommendation, knowledge of herbal medicine, and obtaining health-related information from social media (*n* = 144). Model coefficients (B), odds ratios (OR = Exp(B)), 95% confidence intervals (95% CI), and *p* values are presented in Table 4. Family-based recommendation was independently associated with herbal medicine use (OR = 8.04; 95% CI: 2.99–21.61; *p* < 0.001). Knowledge of herbal medicine was also independently associated with use (OR = 4.56; 95% CI: 1.97–10.58; *p* < 0.001). In contrast, obtaining health-related information through social media was not associated with herbal medicine use (OR = 0.93; 95% CI: 0.35–2.51; *p* = 0.890).

Family-based recommendation and herbal medicine knowledge remained independently associated with herbal medicine use, whereas obtaining information through social media was not. The observed cell frequencies underlying these analyses are presented in Supplementary Table S2.

The multivariable logistic regression model demonstrated satisfactory overall performance. Compared with the null model, the fitted model significantly improved prediction (Omnibus χ^2^ = 42.737, df = 3, *p* < 0.001). Good calibration was observed according to the Hosmer–Lemeshow test (χ^2^ = 0.477, df = 3, *p* = 0.924). The model explained 35.1% of the variance according to the Nagelkerke pseudo-R^2^ and correctly classified 75.7% of participants.

### 3.6. Factors Associated with Recommendation of Herbal Medicine

To identify factors associated with students’ recommending behavior of herbal medicine, bivariate logistic regression analyses were performed using the full study sample (*n* = 144) (Table 5). All predictors were analyzed independently to estimate crude associations with the outcome. Family-based recommendation showed the strongest association with recommendation behavior. Students reporting family-based recommendation were significantly more likely to recommend herbal medicine to others (OR = 4.60; 95% CI: 2.01–10.53; *p* < 0.001). In addition, recommending behavior was associated with higher levels of self-reported knowledge (OR = 2.73; 95% CI: 1.30–5.76; *p* = 0.006), highlighting the role of intergenerational transmission (Table 5). Regarding specific products, prior use of herbal remedies was also significantly associated with recommending behavior, corresponding to increased odds of recommendation (OR = 5.52; 95% CI: 2.44–16.67; *p* < 0.001).

### 3.7. Knowledge, Family Transmission, and Recommendation of Herbal Medicine

The probability that students recommended herbal medicine was evaluated using a multivariable logistic regression model including all predictors identified in the bivariate analysis (Table 6). The model was adjusted for knowledge of herbal medicine, family-based recommendation, prior use of herbal products, and self-reported affiliation with an indigenous community. Model coefficients (B), odds ratios (OR = Exp(B)), 95% confidence intervals (95% CI), and *p* values are presented in Table 6.

Family-based recommendation within the household was independently associated with recommendation behavior. Students who reported that herbal medicine was recommended by family members were significantly more likely to recommend it themselves (OR = 3.70; 95% CI: 1.45–9.44; *p* = 0.006).

Similarly, prior use of herbal products was independently associated with recommending behavior (OR = 4.16; 95% CI: 1.75–9.93; *p* = 0.001).

In contrast, knowledge of herbal medicine (OR = 1.56; 95% CI: 0.67–3.63; *p* = 0.300) and indigenous affiliation (OR = 2.33; 95% CI: 0.47–11.40; *p* = 0.298) were not significantly associated with the outcome. In this sample, the model indicates that recommending behavior is primarily structured by family influence and personal experience rather than by individual knowledge or cultural affiliation.

The recommendation model significantly improved prediction compared with the null model (Omnibus χ^2^ = 22.667, df = 4, *p* < 0.001), with a Nagelkerke pseudo-R^2^ of 0.202 and an overall classification accuracy of 72.2%. However, the Hosmer–Lemeshow test suggested suboptimal calibration (χ^2^ = 10.122, df = 4, *p* = 0.038). Therefore, the estimated associations should be interpreted with appropriate caution.

### 3.8. Social Media Use and Access to Herbal Medicine Information

Because family was identified as the primary source of recommendation, additional analyses were conducted to evaluate whether social media was associated with herbal medicine knowledge among students.

All participants reported using at least one social media platform. Facebook was the most frequently used platform (42.4%), followed by Instagram (20.1%), Snapchat (13.9%), TikTok (12.5%), and X/Twitter (11.1%). Most students (79.2%) reported obtaining health-related information from social media, and 79.9% reported sharing or reacting positively to such content (Table 7).

Bivariate logistic regression analyses were conducted to evaluate the association between digital behaviors and students’ knowledge of herbal medicine (*n* = 144). Actively seeking health-related information through social media was significantly associated with higher odds of reporting knowledge of herbal medicine (OR = 2.97; 95% CI: 1.06–8.27; *p* = 0.038). In contrast, sharing or reacting to content and the use of specific platforms were not significantly associated with knowledge (*p* > 0.05).

A multivariable logistic regression model (Table 8) including all digital variables confirmed that actively seeking health-related information through social media remained significantly associated with knowledge of herbal medicine (OR = 2.97; 95% CI: 1.06–8.27; *p* = 0.038) (Table 8). No significant associations were observed for sharing or reacting to content (OR = 0.47; 95% CI: 0.19–1.18; *p* = 0.106), Facebook use (OR = 0.48; 95% CI: 0.16–1.41; *p* = 0.183), Instagram use (OR = 0.38; 95% CI: 0.11–1.32; *p* = 0.128), Snapchat use (OR = 0.31; 95% CI: 0.07–1.31; *p* = 0.111), or TikTok use (OR = 0.77; 95% CI: 0.19–3.10; *p* = 0.708).

The model assessing factors associated with herbal medicine knowledge showed adequate calibration according to the Hosmer–Lemeshow test (χ^2^ = 2.326, df = 6, *p* = 0.887). However, the overall model was not statistically significant (Omnibus χ^2^ = 9.165, df = 6, *p* = 0.164), explained 8.6% of the variance according to the Nagelkerke pseudo-R^2^, and correctly classified 68.8% of participants. Accordingly, these findings should be interpreted as exploratory.

In this sample, exploratory findings suggest that social media may function primarily as channels for information access and knowledge acquisition rather than as direct determinants of recommending behavior. While digital exposure may contribute to awareness of herbal medicine, recommendation practices appear to be predominantly shaped by interpersonal interactions, particularly family-based transmission. Given that the proportion of use exceeded reported knowledge, the influence of social media may be partially embedded within interpersonal dynamics, as information circulating through digital platforms can be retransmitted through close social networks, reinforcing traditional patterns of knowledge dissemination.

## 4. Discussion

Herbal medicine use was commonly reported among urban adolescents and young adults, supporting previous evidence that these practices remain common despite urbanization and formal education and continue to coexist with biomedical care [6,14,20,50]. Overall, these findings support the view that herbal medicine continues to be incorporated into everyday healthcare practices among younger urban populations despite increasing exposure to biomedical care and formal education.

The finding that all participants who reported herbal medicine use also reported using conventional (allopathic) medical treatment suggests that traditional and biomedical care may coexist within their healthcare experiences. However, because the questionnaire did not distinguish whether both approaches were used simultaneously for the same health condition or at different times for different conditions, this finding should not be interpreted as evidence that herbal medicine and conventional medical treatment were used simultaneously for the same health condition. Rather, it indicates previous experience with both therapeutic approaches [7,13,51]. Nevertheless, this observation has practical implications for healthcare professionals, who should routinely inquire about herbal medicine use to improve patient–provider communication and identify potential herb–drug interactions [52,53]. These discussions are particularly important because herbal products may vary in composition, quality, and dosage, and concomitant use with conventional medications may increase the risk of adverse effects or clinically relevant herb–drug interactions [26,51,54,55,56].

The high prevalence of self-reported knowledge and use observed in this student population aligns with previous ethnomedical research conducted predominantly among adults, patients, or health specialists [18,57]. However, by focusing on adolescents and young adults, this study highlights that such practices are not confined to older generations but continue to be transmitted among younger cohorts, reinforcing the notion of intergenerational continuity rather than cultural rupture [54,55]. This continuity may partly explain why herbal medicine remains culturally relevant even among younger generations living in urban settings.

Family-based recommendation was consistently associated with both herbal medicine use and recommendation behavior, supporting previous evidence that family transmission remains the principal mechanism through which medicinal plant knowledge is maintained across generations, even among urban adolescents and young adults. These findings are consistent with the hypothesis that early-life family experiences continue to influence health-related decision-making [1,58,59].

Notably, recent studies conducted by hospital-based research groups in western Mexico have documented that a significant portion of traditional medicine knowledge circulates not only among patients but also through interactions with healthcare professionals, including physicians and nurses [12]. The present findings complement this evidence by suggesting that healthcare professionals may contribute to the dissemination of traditional medicine knowledge within clinical settings. Together, these findings suggest that although healthcare settings may contribute to knowledge dissemination, family-based transmission remains the dominant mechanism through which medicinal plant knowledge is preserved and translated into health-related behaviors among younger individuals. This observation has practical implications because educational strategies addressing herbal medicine may be more effective when they acknowledge the continuing influence of family networks rather than focusing exclusively on individual-focused educational strategies.

Beyond family-based transmission, digital environments have become increasingly relevant sources of health information among younger populations. A novel aspect of this study was the evaluation of social media as a potential source of herbal medicine information among urban adolescents and young adults. Obtaining health-related information through social media was associated with higher odds of reporting knowledge of herbal medicine, whereas other indicators of digital engagement, such as sharing content or using specific platforms, were not significantly associated.

Although family remained the dominant source of recommendation and the factor most consistently associated with herbal medicine-related behaviors, social media appeared to contribute primarily to information access and self-reported knowledge rather than behavioral change [60,61]. These findings suggest that digital information complements, rather than replaces, traditional family-based knowledge transmission [62]. Information encountered through social media may subsequently be discussed and interpreted within close social networks, allowing digital and interpersonal communication to interact in shaping attitudes toward herbal medicine and subsequent health-related decision-making [63,64].

While the present study does not include phytochemical analyses or biological activity assays, it contributes to ethnobotanical and ethnopharmacological research by documenting the sociocultural pathways through which medicinal plant knowledge is transmitted within urban youth populations. Understanding these transmission mechanisms is essential for contextualizing patterns of use that may later become the focus of pharmacological investigation or drug discovery efforts.

From a clinical perspective, the present findings suggest that healthcare professionals should be aware of the coexistence of herbal medicine use and conventional medical treatment among students when developing culturally appropriate health education strategies. Awareness of family-based transmission, medical pluralism, and digital sources of health information may facilitate communication with young users and support safer, more informed discussions regarding the use of traditional and plant-based products [65,66].

The main strength of this study lies in its focus on an understudied population of urban adolescents and young adults within a sociocultural framework integrating family, healthcare, and digital communication. Nevertheless, several limitations should be acknowledged. The cross-sectional design precludes causal inference, and the use of self-reported measures may have introduced recall and misclassification bias. Although the questionnaire was adapted from previously published instruments and pilot-tested before administration, formal psychometric validation was not performed, and some degree of measurement error cannot be excluded. In addition, the relatively modest sample size limited the number of predictors included in the multivariable models and resulted in wide confidence intervals for some estimates. Although the logistic regression models generally showed acceptable performance, the recommendation model demonstrated suboptimal calibration; therefore, the magnitude and precision of the observed associations should be interpreted with caution. Accordingly, these associations should be confirmed in larger and more diverse populations before stronger inferences can be made. Residual confounding cannot be excluded because, as in any observational cross-sectional study, unmeasured factors may have influenced the observed associations. Finally, because the study was conducted within a single educational setting, the generalizability of the findings may be limited, and future studies in larger and more diverse populations are warranted. Because exposures and outcomes were measured simultaneously, temporal relationships could not be established and reverse causation remains possible.

Future research should incorporate longitudinal designs, more diverse populations, and mixed methods approaches to further elucidate these dynamics. Integrating sociocultural data with ethnobotanical inventories or pharmacological evaluations could further strengthen interdisciplinary links between social science and biomedical research.

## 5. Conclusions

In this sample of urban adolescents and young adults from western Mexico, herbal medicine represented a commonly reported health practice. Family-based recommendation was associated with herbal medicine use and recommendation, whereas social media exposure was associated primarily with knowledge of herbal medicine rather than behavioral outcomes. These findings support incorporating questions about herbal medicine use into routine clinical communication with adolescents and young adults while recognizing the continued importance of family-based knowledge transmission. Given the cross-sectional design and single-center setting, these findings should be confirmed in larger and more diverse populations. These findings provide a foundation for future studies exploring the integration of sociocultural and digital determinants of herbal medicine use among younger populations.

## Figures and Tables

**Table 1:** Sociodemographic and anthropometric characteristics of the study population.

Variable	Category	*n* (%)	Mean ± SD
Sex	Male	66 (45.80)	—
	Female	78 (54.20)	—
Age (years)	All	—	17.99 ± 1.31
	Male	—	17.92 ± 1.29
	Female	—	18.15 ± 1.35
Affiliation to an indigenous community	No	136 (94.40)	—
	Yes	8 (5.60)	—
Employment status	Not working	61 (42.40)	—
	Working	83 (57.60)	—
	Male	37 (44.60)	—
	Female	46 (55.40)	—
Maximum educational level in the household	Illiterate	7 (4.90)	—
	Primary school	4 (2.80)	—
	Middle school	17 (11.80)	—
	High school	57(39.60)	—
	Bachelor’s degree	39 (27.10)	—
	Master’s degree	15 (10.40)	—
	Doctoral degree	5 (3.50)	—
Socioeconomic level (AMAI classification)	E	37 (25.70)	—
	D	34 (23.60)	—
	D+	23 (16.00)	—
	C	23 (16.0)	—
	C+	27 (18.80)	—
	A/B	0 (0.0)	—
Body mass index (kg/m^2^)	All	—	24.61 ± 4.97
	Male	—	24.92 ± 4.40
	Female	—	24.35 ± 5.43

Data are presented as absolute frequencies (n) and percentages (%). Continuous variables are presented as mean ± standard deviation (SD). Indigenous community affiliation refers to self-reported identification with or belonging to an indigenous group. Socioeconomic level was assessed according to the Mexican Association of Market Intelligence and Opinion Agencies (AMAI) classification, categorized as: A/B (high), C+ (upper-middle), C (middle), D+ (lower-middle), and D/E (low). Dashes (—) indicate non-applicable data.

**Table 2:** Characteristics of herbal medicine use among students who reported prior use (*n* = 53).

Variable	Category	*n* (%)
Source of recommendation	Family	32 (60.4)
	Friends	3 (5.7)
	Herbalists (hierberos)	4 (7.5)
	Neighbors	2 (3.8)
	Physicians/nurses	4 (7.5)
	Complementary medicine practitioner	4 (7.5)
	Other	4 (7.5)
Reason for use	Muscular pain	13 (24.5)
	Stomach problems	5 (9.4)
	Cancer	6 (11.3)
	Hypertension	3 (5.7)
	High lipids/high cholesterol	5 (9.4)
	Weight control (loss/gain)	6 (11.3)
	Others	15 (28.3)
Frequency of use	Once per week	39 (73.6)
	Three times per week	6 (11.3)
	Daily use	8 (15.1)

Data are presented as absolute frequencies (*n*) and percentages (%). Knowledge of HM refers to student self-reported understanding of the concept. Sources of recommendation, reasons for use, and frequency of use correspond exclusively to participants who reported previous herbal medicine use (*n* = 53). Categories translated from Spanish preserve local terminology (e.g., hierberos) to maintain ethnobotanical contextual accuracy. Person providing the recommendation (social source) refers to the social actor who suggested the remedy, not to documentary or institutional information sources.

**Table 3:** Bivariate associations between selected factors and herbal medicine use (*n* = 144).

Variable	Comparison	OR	95% CI	*p* Value
Family-based recommendation	Yes vs. No	10.71	4.18–27.45	<0.001
Knowledge of herbal medicine	Yes vs. No	6.11	2.84–13.15	<0.001
Obtains information from social media	Yes vs. No	1.21	0.52–2.83	0.658

OR expresses crude odds of herbal medicine use (dependent variable = 1). Associations were evaluated using Pearson’s χ^2^ test. Odds ratios (OR) and 95% confidence intervals (CI) were calculated from 2 × 2 contingency tables. All expected cell counts were ≥5; therefore, Fisher’s exact test was not required. Values in bold indicate statistically significant associations.

**Table 4:** Multivariable logistic regression model for factors associated with herbal medicine use (*n* = 144).

Variable	Category	B	OR (Exp(B))	95% CI	*p* Value
Family-based recommendation	Yes vs. No	2.085	8.04	2.99–21.61	<0.001
Knowledge of herbal medicine	Yes vs. No	1.517	4.56	1.97–10.58	<0.001
Obtains health information from social media	Yes vs. No	−0.070	0.93	0.35–2.51	0.890

OR expresses adjusted odds of herbal medicine use. All variables were entered simultaneously using the enter method. Values in bold indicate statistically significant associations.

**Table 5:** Bivariate logistic regression analysis of factors associated with students’ recommendation of herbal medicine.

Variable/Comparison	OR	95% CI	*p* Value
Knowledge of herbal medicine (yes vs. no)	2.73	1.30–5.76	0.006
Family-based recommendation (yes vs. no)	4.60	2.01–10.53	<0.001
Prior use of herbal medicine (yes vs. no)	5.52	2.44–16.67	<0.001
Indigenous affiliation (yes vs. no)	1.53	0.32–7.26	0.596

OR expresses crude odds of recommending herbal medicine. Each predictor was analyzed independently using binary logistic regression.

**Table 6:** Multivariable logistic regression model for factors associated with students’ recommendation of herbal medicine (*n* = 144).

Predictor	B	OR (Exp(B))	95% CI	*p* Value
Knowledge of herbal medicine (yes)	0.446	1.56	0.67–3.63	0.300
Family-based recommendation (yes)	1.309	3.70	1.45–9.44	0.006
Prior use of herbal products (yes)	1.426	4.16	1.75–9.93	0.001
Indigenous affiliation (yes)	0.844	2.33	0.47–11.40	0.298

OR expresses the odds that students recommended herbal medicine to others (dependent variable = 1). Predictors were entered using the enter method. Values in bold indicate statistically significant associations.

**Table 7:** Use of social media and participation in the diffusion of information on herbal medicine.

Variable	Category	*n* (%)
Most frequently used social media platform	Facebook	61 (42.4)
	Instagram	29 (20.1)
	Snapchat	20 (13.9)
	TikTok	18 (12.5)
	X (Twitter)	16 (11.1)
Obtains information from social media about herbal medicine	No	30 (20.8)
	Yes	114 (79.2)
Has shared or reacted positively to content on herbal medicine	No	29 (20.1)
	Yes	115 (79.9)

Values are presented as number of students (n) and percentage (%). Participants were asked which social media platform they used most frequently and whether they had obtained, shared, or reacted to content related to herbal medicine.

**Table 8:** Multivariable logistic regression model for factors associated with knowledge of herbal medicine (*n* = 144).

Variable	Category	B	OR (Exp(B))	95% CI	*p* Value
Obtains health-related information from social media	Yes vs. No	1.087	2.97	1.06–8.27	0.038
Has shared or reacted positively to herbal medicine content	Yes vs. No	−0.757	0.47	0.19–1.18	0.106
Uses Facebook	Yes vs. No	−0.733	0.48	0.16–1.41	0.183
Uses Instagram	Yes vs. No	−0.969	0.38	0.11–1.32	0.128
Uses Snapchat	Yes vs. No	−1.188	0.31	0.07–1.31	0.111
Uses TikTok	Yes vs. No	−0.267	0.77	0.19–3.10	0.708

Values are presented as regression coefficients (B), odds ratios (OR), 95% confidence intervals (CI), and *p* values. OR expresses adjusted odds of reporting knowledge of herbal medicine. All variables were entered simultaneously using the enter method. Values in bold indicate statistically significant associations.

## Data Availability

The original contributions presented in the study are included in the article/Supplementary Materials; further inquiries can be directed to the corresponding authors.

## Supplementary Materials

The following supporting information can be downloaded at https://www.mdpi.com/article/10.3390/healthcare14142161/s1, Table S1: Questionnaire items used for data collection and variable operationalization. Table S2: Observed cell frequencies underlying the multivariable logistic regression models.
