# Supplementary material for: Knowledge and Use of Herbal Medicine Among Urban Adolescents and Young Adults in Western Mexico: Family Transmission, Social Media Exposure, and Associated Factors
Source: Healthcare (Basel). 2026 Jul 17;14(14):2161. doi: 10.3390/healthcare14142161 (PMC13410035; doi:10.3390/healthcare14142161)
Supplement: Supplementary file 1 [file healthcare-14-02161-s001.zip › healthcare-4392095-supplementary.pdf]

Supplementary Materials

Table S1. Questionnaire items used for data collection and variable operationalization.

Knowledge and Use of Herbal Medicine among Urban Adolescents and Young Adults in Western Mexico: Family Transmission, Social Media Exposure, and Associated Factors

Instructions: The following questionnaire includes only the items analyzed in the present study. These questions were extracted from a broader institutional survey on health-related behaviors conducted among upper-secondary students.
Participation was voluntary and anonymous. Participants were instructed to answer all questions honestly.

Section A. Sociodemographic Characteristics
A1. Age
_____ years

A2. Sex
☐ Male
☐ Female

A3. Are you currently employed?
☐ Yes
☐ No

A4. Do you identify yourself or your family as belonging to an Indigenous community?
☐ Yes
☐ No

A5. Highest educational level attained by any member of your household
☐ Illiterate
☐ Primary school
☐ Middle school
☐ High school
☐ Bachelor's degree
☐ Master's degree
☐ Doctoral degree

A6. Highest educational level completed by the head of the household
☐ No formal education
☐ Primary school
☐ Secondary school
☐ High school
☐ Technical degree
☐ Bachelor's degree
☐ Postgraduate degree

A7. Number of complete bathrooms in the household
☐ 0
☐ 1
☐ 2
☐ 3 or more

A8. Number of automobiles owned by the household
☐ 0
☐ 1
☐ 2
☐ 3 or more

A9. Does your household have internet access?
☐ Yes
☐ No

A10. Number of bedrooms in the household

☐ 1
☐ 2
☐ 3
☐ 4 or more

Section B. Knowledge of Herbal Medicine

B1. Do you know what herbal medicine is?
☐ Yes
☐ No

B2. Have you ever used herbal medicine?
☐ Yes
☐ No

B3. How often do you use herbal medicine?
☐ Once per week
☐ Three times per week
☐ Daily
☐ Occasionally

Section C. Reasons for Herbal Medicine Use

If you have used herbal medicine, for what purpose?
(Check all that apply.)
☐ Digestive disorders
☐ Respiratory symptoms
☐ Musculoskeletal pain
☐ Dermatological conditions
☐ Relaxation or sleep
☐ Diabetes
☐ Hypertension
☐ High cholesterol
☐ Weight control
☐ Cancer-related purposes
☐ Immune system support
☐ Menstrual disorders
☐ Other: ___________________

Section D. Source of Recommendation

D1. Who recommended the use of herbal medicine to you?
(Select the main source of recommendation.)
☐ Family member
☐ Friend
☐ Neighbor
☐ Traditional healer (herbalist)
☐ Physician
☐ Nurse
☐ Pharmacist
☐ Complementary medicine practitioner
☐ Social media
☐ Internet websites
☐ Other: ___________________

Section E. Recommendation Practices

E1. Have you ever recommended herbal medicine to another person?
☐ Yes
☐ No

Section F. Concurrent Use with Conventional Medicine

F1. Have you ever used herbal medicine together with conventional (allopathic) medical treatment?
☐ Yes
☐ No

If Yes, please answer the following questions:

F2. Was the conventional treatment used for the same health condition for which you used herbal medicine?

☐ Yes
☐ No
☐ Do not remember
F3. Which conventional treatment(s) did you use? *(Check all that apply.)*
☐ Antibiotics
☐ Analgesics/anti-inflammatory drugs
☐ Antipyretics
☐ Antihypertensive medication
☐ Antidiabetic medication
☐ Gastrointestinal medication
☐ Respiratory medication
☐ Dermatological medication
☐ Other: ___________________

F4. Was the conventional medication:
☐ Prescribed by a physician
☐ Recommended by a pharmacist
☐ Self-medicated (without prescription)
☐ Other: ___________________

F5. For approximately how long did you use both herbal medicine and conventional treatment?
☐ Less than 1 week
☐ 1–4 weeks
☐ 1–3 months
☐ More than 3 months
☐ Do not remember
F6. Did you inform a healthcare professional that you were also using herbal medicine?
☐ Yes
☐ No

Section G. Herbal Medicine Categories
G1. Which types of herbal medicine have you used?
(Check all that apply.)
☐ Digestive herbs
☐ Respiratory/expectorant herbs
☐ Relaxant or sedative herbs
☐ Dermatological herbs
☐ Anti-inflammatory or wound-healing herbs
☐ Hypoglycemic/antidiabetic herbs
☐ Immunostimulant herbs
☐ Emmenagogue herbs
☐ Other: ___________________

Section H. Social Media and Digital Health Information
H1. Which social media platform do you use most frequently?
(Select one.)
☐ Facebook
☐ Instagram
☐ Snapchat
☐ TikTok
☐ X (formerly Twitter)
☐ Other: ___________________

H2. Have you ever obtained information about herbal medicine through social media?
☐ Yes
☐ No

H3. Have you ever shared, liked, commented on, or otherwise reacted to content related to herbal medicine on social media?
☐ Yes
☐ No

H4. Approximately how often do you use social media?

☐ Less than once a week
☐ 1–3 days per week
☐ 4–6 days per week
☐ Every day

H5. Which type of information have you searched for on social media? *(Check all that apply.)*

☐ Medicinal plants
☐ Herbal remedies
☐ Traditional medicine
☐ Home remedies
☐ Disease treatment
☐ Healthy lifestyle
☐ Other: ___________________

H6. Which of the following best describes your interaction with herbal medicine content on social media? *(Check all that apply.)*

☐ I only read or view the information
☐ I like or react to posts
☐ I comment on posts
☐ I share posts
☐ I discuss the information with family or friends
☐ I have used herbal medicine based on information found on social media
☐ None of the above

Section I. Knowledge Transmission

I1. From which sources have you obtained information or knowledge about herbal medicine? *(Check all that apply.)*

☐ Family members
☐ Friends
☐ Neighbors
☐ Traditional healers (herbalists)
☐ Healthcare professionals (physicians, nurses, pharmacists)
☐ School
☐ Internet websites
☐ Social media
☐ Television
☐ Books or magazines
☐ Other: ___________________

This supplementary table contains the questionnaire items analyzed in the present study. These items were extracted from a broader institutional survey on health-related behaviors conducted among upper-secondary students. The questionnaire was adapted from previously published instruments and supplemented with study-specific items addressing family transmission, concurrent use with conventional medicine, and social media exposure.

Table S2. Observed cell frequencies underlying the multivariable logistic regression models.

Outcome	Predictor	Category	No	Yes	Total
**Current herbal product use**	Previous herbal medicine use	No	78	13	91
		Yes	25	28	53
**Recommendation of herbal medicine**	Family-based recommendation	No	84	28	112
		Yes	11	21	32
	Knowledge of herbal medicine	No	72	23	95
		Yes	26	23	49
**Knowledge of herbal medicine**	Obtains health-related information through social media	No	24	6	30
		Yes	74	40	114

Observed cell frequencies corresponding to the predictors included in the multivariable logistic regression models. For each outcome, Yes indicates the presence of the outcome (current herbal product use, recommendation of herbal medicine, or knowledge of herbal medicine), whereas No indicates its absence. These data are provided to improve transparency and facilitate the interpretation of the reported odds ratios.
